# Safety evaluation of PEGylated MNPs and p-PEGylated MNPs in SD rats

**DOI:** 10.1038/s41598-023-48742-w

**Published:** 2023-12-06

**Authors:** Hairuo Wen, Guitao Huo, Chao Qin, Hui Wu, Dan Wang, Mo Dan, Xingchao Geng, Shujie Liu

**Affiliations:** 1 National Center for Safety Evaluation of Drugs, Key Laboratory of Beijing for Nonclinical Safety Evaluation Research of Drugs, National Institutes for Food and Drug Control, Beijing, 100176 People’s Republic of China; 2 China Pharmaceutical University, Nanjing, 211198 Jiangsu Province People’s Republic of China; 3 State Key Laboratory of Novel Pharmaceutical Preparations and Excipients, CSPC Pharmaceutical Group Co., Ltd., Shijiazhuang, 050035 Hebei People’s Republic of China; 4 Center for Drug Evaluation, National Medical Products Administration, Beijing, 100022 People’s Republic of China

**Keywords:** Drug safety, Toxicology, Nanomedicine, Nanoscale materials, Nanotoxicology

## Abstract

Polyethylene glycol-coated magnetic nanoparticles (PEGylated MNPs) have demonstrated prominent advantages in cancer diagnosis and hyperthermia therapy. However, there is currently lack of standard mode and sufficient toxicity data for determining the delayed risk of PEGylated MNPs. Nevertheless, the toxicity potentials, especially those associated with the oxidative stress, were ubiquitously reported. In this study, PEGylated MNPs and p-PEGylated MNPs were administrated to SD (Sprague Dawley) rats by single intravenously injection, and various toxicity indicators were monitored till 56 days post-administration for a comprehensive toxicity evaluation. We revealed that both nanoparticles could be rapidly cleared from plasma and enter tissues, such as, liver, kidneys and spleen, and p-PEGylated MNP is less prone to be accumulated in the tissues, indicating a lower toxicity risk. PEGylated MNPs were more likely to up-regulate the expression levels of Th2 type cytokines and trigger inflammatory pathways, but no related pathological change was found. Both MNPs are not mutagenic, while recoverable mild DNA damage associated with the presence of nanoparticles might also be observed. This study demonstrated a research approach for the non-clinical safety evaluation of nanoparticles. It also provided comprehensive valuable safety data for PEGylated and p-PEGylated MNPs, for promoting the clinical application and bio-medical translation of such MNPs with PEG modifications in the cancer diagnosis and therapy.

## Introduction

Magnetic resonance imaging (MRI) has offered prominent advantages in soft tissue resolution and tissue penetration and has been widely used in cancer diagnosis as a non-invasive and radiation-free technique^1^. For a high-quality imaging, contrast agents with low toxicity and high relaxation properties have become the focus of research and development^2^. The good biocompatibility of iron endows magnetic nanoparticles (MNPs) a hotspot for MRI contrast agents, and the magnetocaloric effect of iron oxide could effectively combine MRI with hyperthermia therapy^3^. Till now, the magnetic MNPs are the only nanomaterials approved by the FDA for cancer diagnosis, cancer hyperthermia therapy, and iron deficiency anemia. Among which, polyethylene glycol(PEG)-ylation, which could reduce the phagocytosis of nanoparticles by the reticuloendothelial system (RES), prolong the circulation time and increase their accumulation in tumors, has thereby become the most popular modification for the MNPs. Many studies have previously demonstrated the benefits of PEG-coated MNPs in the applications of MRI. For instance, PEG coated super-paramagnetic MNPs showed as a long-lasting MRI effects in the brain of mice, with a relatively short imaging time period^4^. In addition, low RES retention and high urinary excretion have been recognized as the properties of PEG-copolymer-coated MNPs of ~ 20 nm. These nanoparticles were accumulated in proximal tubule cells and made them feasible as novel MRI contrast agents for kidneys^5^. Our previous study has demonstrated the PEG-coated Fe_3_O_4_ nanoparticles would be benefit in both MRI and SPECT as a sensitive approach for tumor detection^6^. On another hand, MNPs were suggested to be agents for thermal and immune therapies in cancers. As reported by Zanganeh et al.^7^ Ferumoxytol, an iron nanoparticle compound, could predominantly inhibit the proliferation of tumor cells in the mice, and the therapeutic effect was attributed to the polarization of macrophage into a pro-inflammatory M1 phenotype.

Concerns on the risk of nanoparticles to human health come along with their prospects in biomedical fields. The safety of MNPs has been proved by its wealth of clinical experience, whereas their toxicity potentials, especially those associated with the oxidative stress, were ubiquitously reported. Zhu et al. suggested that Fe_2_O_3_ and Fe_3_O_4_ nanoparticles could trigger the oxidative stress injury and apoptosis in endothelial cells, and consequently increase the risk of cardiovascular diseases^8,9^ revealed that iron nanoparticles at 20 ~ 80 nm could down-regulated the expression of genes for pro-inflammatory response, human bronchial epithelial cells, despite no significant oxidative injury was induced^9^. Furthermore, the Fe_2_O_3_ nanoparticles could pass through the alveolar capillary barrier of rats and distributed to liver, spleen, kidneys, and testicles, where the mononuclear phagocytes are gathered^10^. Salimi et al. studied the toxicity of MNPs in Balb/c mice and suggested that the MNPs at 10 mg/kg could elevate the levels of blood urea nitrogen and direct bilirubin^11^. Moreover, edema and losing cytoplasm was found in the liver, while apoptotic cells were observed in the heart of mice. Compelling evidence have demonstrated that reactive oxidative stress could activate p53 at different degrees to bidirectionally regulate autophagy and promote apoptosis of cells^12^. These literatures drew our attention to the toxicity potentials of iron nanoparticles in lungs and accumulated systematically.

Nanoparticles differ from traditional pharmaceutical bioproducts in the way they interact with cells and tissues^13^. Risks on the immunotoxicity and genotoxicity have become the major safety concerns of nanotoxicities. For example, Di Gioacchino et al. suggested that the pro-inflammatory effects of nanoparticles in the lungs, as IL-1beta, MIP-1alpha, MCP-1, MIP-2 were increased, and MAPKs p38 and JNKs pathways could be activated^14^. MNPs could produce immunotoxicity by mediating the functions of multiple immune cells^15^, including monocytes, macrophages, lymphocytes, and dendritic cells. Ansari et al. revealed that Fe_3_O_4_ nanoparticles of ~ 60 nm was able to interact with DNA and form a complex between the base pairs of lymphocytes^16^, and structural chromosomal aberrations as well as micronucleus formation in the bone marrow cells induced by the nanoparticles were observed.

Despite the great clinical application potential, the in vivo toxicity of MNPs has not been fully and comprehensively investigated. In this study, the toxicity risks of two PEG-coated MNPs synthesized by our group were comprehensively evaluated from the perspectives of tissue distribution, immunotoxicity, organ toxicity and genotoxicity. Our results indicated the safety of PEG-coated MNPs and shed lights on their application and transformation.

## Materials and methods

### Chemicals

Iron (III) acetylacetonate (Fe(acac)3) was purchased from Aldrich (14,024-18-1) and used after 2 times recrystallizations. Oleylamine was purchased from Fluka (112-90-3, g70%), and oleic acid was purchased from Sigma-Aldrich (112-80-1) and both used as received. HOOC-PEG-COOH was synthesized according to literature^17^. Analytical grade chemicals such as ethanol, ether, and diphenyl oxide were purchased from Sinopharm Chemical Reagent Beijing, Co., Ltd. Diphenyl oxide was used after further purification by reduced pressure distillation. Other chemicals of analytical grade including ethanol, dichloromethane, and ether were used as received.

### Preparation of PEGylated MNPs and p-PEGylated MNPs

PEGylated MNPs: Fe_3_O_4_nanoparticles were prepared as previously described^18^. Typically, 2.12 g of Fe(acac)3, 7.90 mL of oleylamine, and 24.0 g of HOOC-PEG-COOH were dissolved in 100 mL of diphenyl oxide solution. After being purged with nitrogen for 2 h, the solution was heated to reflux within 15 min. Under mechanical stirring at 400 rpm, the heating process with reflux maintained for 2 h and then the reaction system was cooled to room temperature. Upon addition of a mixture of ethanol and ether (vol:vol = 1:5) into the aliquots at room temperature, the resultant nanocrystals were precipitated and isolated. By being redispersed in ethanol and subsequently precipitated with ether for three cycles, the nanocrystals were purified and collected for further characterizations.

p-PEGylated MNPs: Fe_3_O_4_ nanoparticles with core size of 3.6 nm were synthesized as previously described with slight modifications^19^. Typically, 1.41 g of Fe(acac)3, 3.39 g of oleic acid, 3.21 g of oleylamine, and 2.70 g of 1-octadecanol were dissolved in 40 mL of diphenyl ether. After being purged with nitrogen for 30 min, the solution was refluxed for 30 min under stirring. Then the reaction system was cooled to room temperature. The resultant nanoparticles were precipitated by ethanol, collected by centrifugation, washed with ethanol for three times, and finally re-dispersed in THF or cyclohexane for further experiments.

As a typical example, 150 mg of PEG derivative was dissolved in 10 mL of THF containing 10 mg hydrophobic Fe_3_O_4_ nanoparticles. Subsequently, the reaction mixture was heated to 60 °C and kept at this temperature for 12 h under stirring. After that, the Fe_3_O_4_ nanoparticles were precipitated by cyclohexane, washed with cyclohexane for three times, and then dried under vacuum at room temperature. The particle powders obtained, independent of the anchoring group of PEG derivatives and the particle core size, were found to be readily dissolved in water, supporting that the PEG coating was effectively realized. To remove excess PEG ligand, the resultant aqueous solutions containing the PEGylated Fe_3_O_4_ nanoparticles were purified by ultrafiltration for 4 cycles using 100 kDa MWCO centrifugal filter (Millipore YM-100).

### Characterization

Transmission electron microscope (TEM) images of the nanocrystals were taken on a JEM-100CXII electron microscope at an acceleration voltage of 100 kV. Dynamic light scattering (DLS) measurements were carried out at 298.0 K with a Nano ZS (Malvern) equipped with a solid-state He_Ne laser (λ = 633 nm) for measuring the hydrodynamic size of the resultant nanoparticles.

### Animals and administration

A total of 48 male Sprague–Dawley (SD) rats, at about 6 weeks old of age, were purchased from Beijing Vital River Laboratory Animal Technology Co. Ltd (Beijing, China). All animals were housed in the barrier system and maintained in polycarbonate mouse boxes at 20 to 25 °C with 40% to 70% relative humidity, a 12-h light–dark cycle. The rats were given free access to tap water and diet ad libitum.

The animals were quarantined for 5 days and subsequently randomized into 4 groups according to the body masses, which are Control Group (Ctl), PEGylated MNPs Group (PEGylated MNPs), p-PEGylated MNPs Group (p-PEGylated MNPs), and Ethylnitrosourea Positive Group (ENU), with 12 males per group. The rats were intravenously administrated with 0.9% NaCl injection, 10 mg/kg of PEGylated MNPs, and 10 mg/kg of p-PEGylated MNPs respectively by a single dose, while the 40 mg/kg of ENU was intragastrically given to the rats by a single dose as well. The study is presented in accordance with Animal Research: Reporting of In Vivo Experiments (ARRIVE) guidelines.

### Plasma and tissue distribution determination

Peripheral blood was collected 5 min, 1 h, 3 h, 6 h, 24 h, 48 h,7 days, 21 days, 28 days, 42 days and 56 days post-administration, and tissues, includes, heart, lungs, brain, liver, kidneys, spleen, thymus, testes, and epididymides were collected on 14 days and 56 days post-administration. These samples were digested in a mixture of concentrated hydrochloric and nitric acid (1:3) using the microwave digestion system. The contents of both PEGylated MNPs and p-PEGylated MNPs in different tissues were detected through inductively coupled plasma–mass spectrometry (NexION 300X, Perkin Elmer, U.S.A). The MNPs in the plasma or tissues were treated with nitric acid. All samples were subsequently digested by a microwave digestion instrument (MARS, CEM, Matthews, NC, USA). The concentration of Fe in prepared standards and samples were measured by inductively coupled plasma–mass spectrometry (ICP-MS, NexION 300X, Perkin Elmer, USA).

### General toxicity

**Figure 1:**
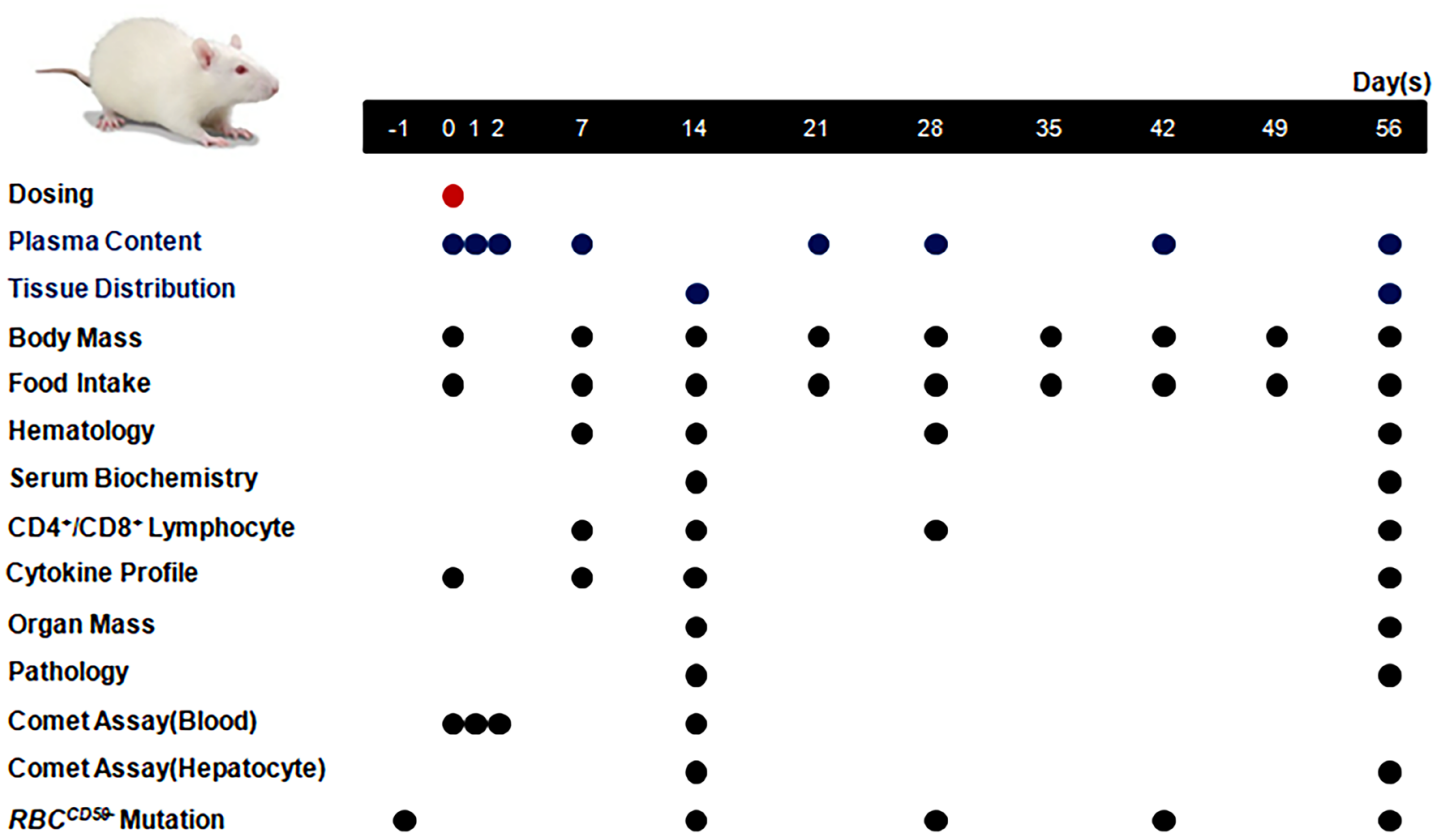
Study Schedule. Plasma content of PEGylated MNPs and p-PEGylated MNPs 5 min, 1 h, 3 h, 6 h, 24 h, 48 h, 7 days, 21 days, 28 days, 42 days and 56 days post-administration, and tissues, includes, heart, lungs, brain, liver, kidneys, spleen, thymus, testes, and epididymides were collected on 14 days and 56 days post-administration. Clinical symptoms were observed every day, while animal body mass and food intake were measured every week. Hematology and CD4^+^/CD8^+^ lymphocyte determination were performed on 7,14,28 and 56 days post-administration, the biochemical detection were performed on 14 and 56 days post-administration, and the cytokine profiles of animals were detected on 0, 7,14 days, and 56 days post-administration. Animals were anesthetized on 14 days and 56 days post-administration respectively for necropsy and histopathological examination. Comet assay was performed on 3 h, 24 h, 48 h and 14 days post-administration for the peripheral blood, and 14 days and 56 days post-administraion, respectively. *Pig-a* gene mutation for RBC^CD59-^ were performed before the administration, and on 14, 28, 42 and 56 days post-administration, respectively.

Subsequent to dosing, all animals were kept for 2 or 8 weeks for observation and sampling. Clinical symptoms were observed on daily basis, and the body masses and food intakes of animals were measured once per week. Blood samples of about 0.7 mL were collected in K_2_EDTA treated tube on 7 days, 14 days, 28 days, and 56 days subsequent to administration for hematological and T lymphocyte subsets counting respectively. Parameters for hematological examinations, including count of leukocytes (WBC), neutrophils (NEUT), lymphocytes (LYMPH), monocytes (MONO), eosinophils (EOS), basophils (BASO), erythrocytes (RBC), platelets (PLT) and reticulocytes (#Retic) were determined using ADVIA2120i hematology system (Bayer Health Care Co., Germany) and the percentage of CD3^+^/CD4^+^ and CD3^+^/CD8^+^ in total CD3^+^ lymphocytes were determined using FACSCalibur flow cytometry (BD Biosciences, U.S.A). About 1.5 mL of blood per animal was collected for serum biochemical examination on 14 days and 56 days after dosing, and the levels of glutamic-pyruvic transaminase (ALT), aspartic acid aminotransferase (AST), alkaline phosphatase (ALP), creatine phosphokinase (CK), lacticdehydrogenase (LDH), total bilirubin (TBIL), and urea nitrogen (UREA), creatinine (CRE), total protein (TP), albumin (ALB), and albumin/globublin(A/G) were determined using the 7180 Biochemistry Automatic Analyzer (Hitachi Ltd, Gyeonggi-do, South Korea).In addition, 0.2 mL of peripheral blood per animal was taken 6 h, 7 days, 14 days and 56 days after dosing for detecting the changes on cytokine profiles (IL-1α, IL-1β,IL-2, IL-4, IL-6, IL-10, IL-12p70, TNFα and VEGF) in rats using Rat Expanded Cytokine Magnetic (RECYTMAG-65 K-02, Millipore Corp., U.S.A).Half of the animals per group were sacrificed on 14 days post-administration, while the rest 6 animals per group were sacrificed on 56 days post-administration. The masses of heart, lungs, brain, liver, spleen, kidneys, thymus, adrenal glands, testes, and epididymides of animals were measured during necropsy. All these tissues were fixed in a 10% formalin solution and embedded in paraffin, the fixed samples were cut into sections of about 3 ~ 5 μm and stained with hematoxylin and eosin(H&E) for histopathologic examination by light microscopy. See Fig. 1 for the study schedule.

### Comet assay

To determine the DNA damage potential of the PEGylated MNPs and p-PEGylated MNPs, peripheral blood samples of 50 μL (in K_2_EDTA microtainer tube) were collected at 3 h ,24 h, 48 h and 14 days, and hepatocytes collected on 14 days and 56 days after administration were prepared for comet assay. Approximate 50 μL of single cell suspension (around 1 × 10^5^ live cells/mL) were mixed with 500 μL low melting agarose in 37℃ water bath, and 50 μL of the mixtures were placed onto the slides. The slides were placed at 4 °C in the dark for no less than 10 min, and subsequently transfer to the pre-cooled lysis buffer at 4 °C overnight. The slides were placed into the electrophoresis tank and immersed in the cold electrophoresis buffer (containing 200 mM NaOH and 1 mM EDTA, pH > 13) for 20 min for DNA unwinding, and the electrophoresis was performed subsequently with a power supply maintained at 0.7 V/cm (~ 300 mA) for 20 min. The slides were rinsed with neutralization buffer (0.4 M Tris-base, pH7.4), followed by a dehydration with 95% ethanol for 20 min. The prepared slides were air dried, and stained with diluted (1:10,000) SYBR® Green I Nucleic Acid Gel Stain (Invitrogen, S7563) for 5 min at room temperature in the dark. At least 100 cells per sample were independently scored using fluorescent microscope (Nikon eclipse 80i, excited at ~ 497 nm and the emitted wavelength was collected at ~ 520 nm), and Comet Assay IV (Instem, Staffordshire, UK) was used to analyze the medium value of percent (%) tail DNA in each sample.

### Mutation detection on RBC^CD59-^

To determine the DNA mutation potential, blood samples were collected on 1 day before dosing, and the 14, 28, 42, and 56 days after dosing, and were analyzed as previously described by Wang et al.^20^ Approximately 1.5 µL of blood sample for each animal was transferred to a tube containing 200 μL PBS, 2.5 µL anti-CD45-PE (BD Biosciences, CA, USA), and 1 µL anti-CD59-FITC (BD Biosciences, CA, USA). The mixtures were incubated in dark for 30 min at room temperature, and centrifuged for 5 min at 1,000 rpm subsequently. The cell pellets were harvested and resuspended using 0.5 mL PBS, and the incidence of erythrocytes (RBC)^CD59-^ and reticulocytes (RET)^CD59-^ was detected using the FACS Calibur (BD Biosciences, CA, USA).

### Statistics

All data were presented as mean ± standard deviation (SD) and analyzed using GraphPad Prism 8 software (GraphPad Software, Inc., La Jolla, USA). Inter-group variations were calculated by One-Way ANOVA, and the difference was considered as statistically significant when p-values are less than 0.05 (*p* < 0.05).

### Ethical Statement

In this study, all animal experiments and sample collections were performed within the barrier system at the National Center for Safety Evaluation of Drugs (NCSED) in accordance with ARRIVE guidelines. The protocols were approved by the Institutional Animal Care and Use Committee at NCSED (IACUC-2017-K017) and conducted with the IACUC Constitution of NCSED, the Guide for the Care and Use of Laboratory Animals (https://grants.nih.gov/grants/olaw/Guide-for-the-Care-and-use-of-laboratory-animals.pdf) and AAALAC International’s Position Statement. The animals were anaesthetized by isoflurane (Heibei Yipin Pharmaceutical Co., Ltd) by inhalation (small animal anesthesia machine, RWD Life Science Co., Ltd, R520IP) before blood sampling or necropsy. There was no obvious abnormal symptom observed in all the animals during the study. All study protocols (including the research question, key design features, and analysis plan) were prepared before the study and archived at NCSED.

### Originality statement

All the figures and tables in this article have not been published previously, and not under consideration for publication elsewhere, in whole or in part.

## Results

### Characterization

**Figure 2:**
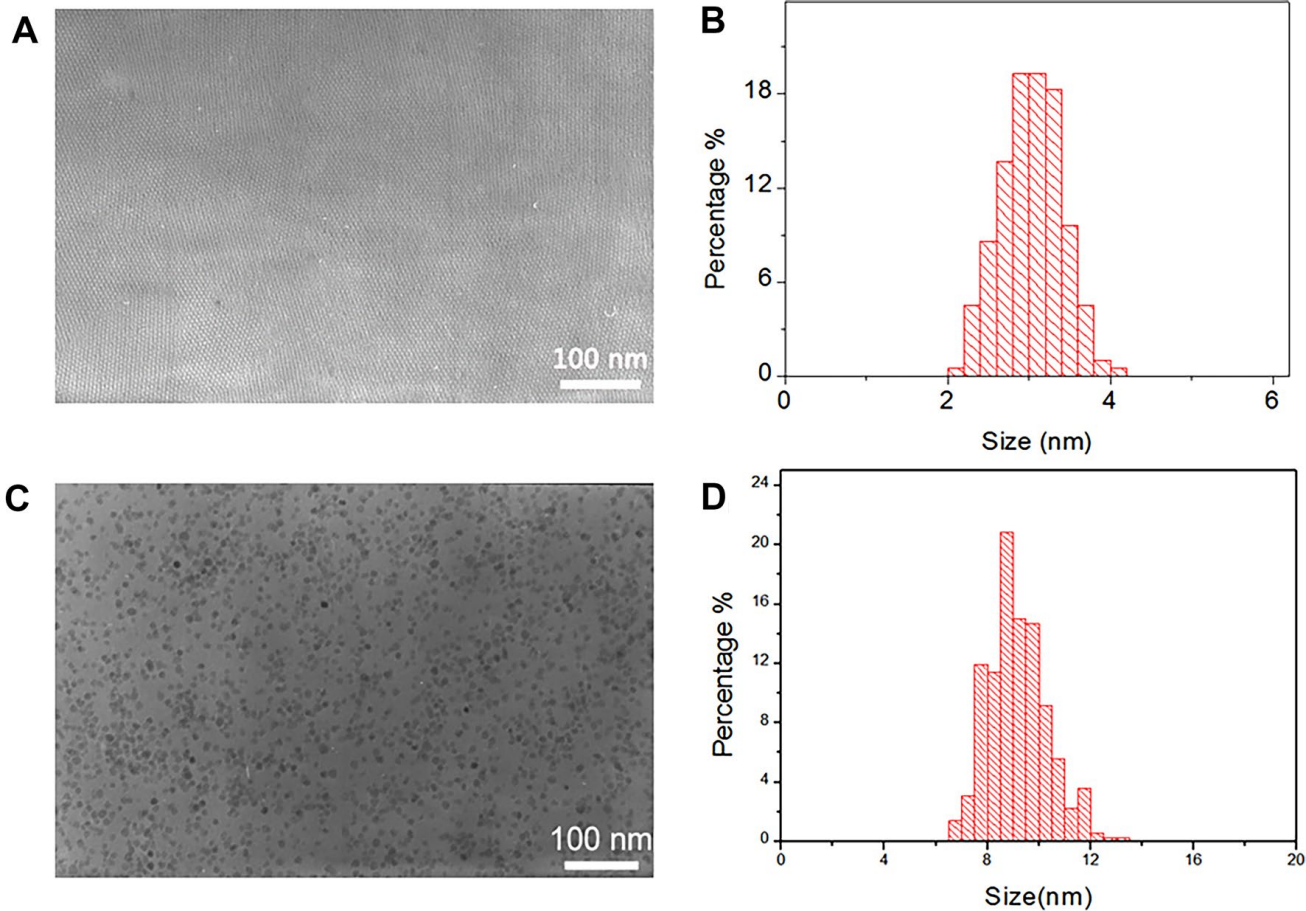
Characteristics of PEGylated MNPs and p-PEGylated MNPs. Characteristics of PEGylated MNPs and p-PEGylated MNPs: TEM image (**A**) and size distribution (**B**) of p-PEGylated MNPs TEM image (**C**) and size distribution (**D**) of PEGylated MNPs. Careful statistical studies show that the size of the PEGylated MNPs and p-PEGylated MNPs are 9.2 nm and 3.0 nm typically.The scale bars represent 100 nm.

The TEM images of the as-prepared nanoparticles obtained are presented (Fig. 2). Careful statistical studies show that the size of the PEGylated MNPs and p-PEGylated MNPs are 9.2 nm and 3.0 nm typically. Further dynamic light scattering (DLS) shown that the Z-Average diameter for the PEGylated MNPs and p-PEGylated MNPs are 63.7 nm and 41.8 nm in water, and the Zeta potentials are 31.1 mV and 1.14 mV.

### Biodistribution of PEGylated MNPs and p-PEGylated MNPs

**Figure 3:**
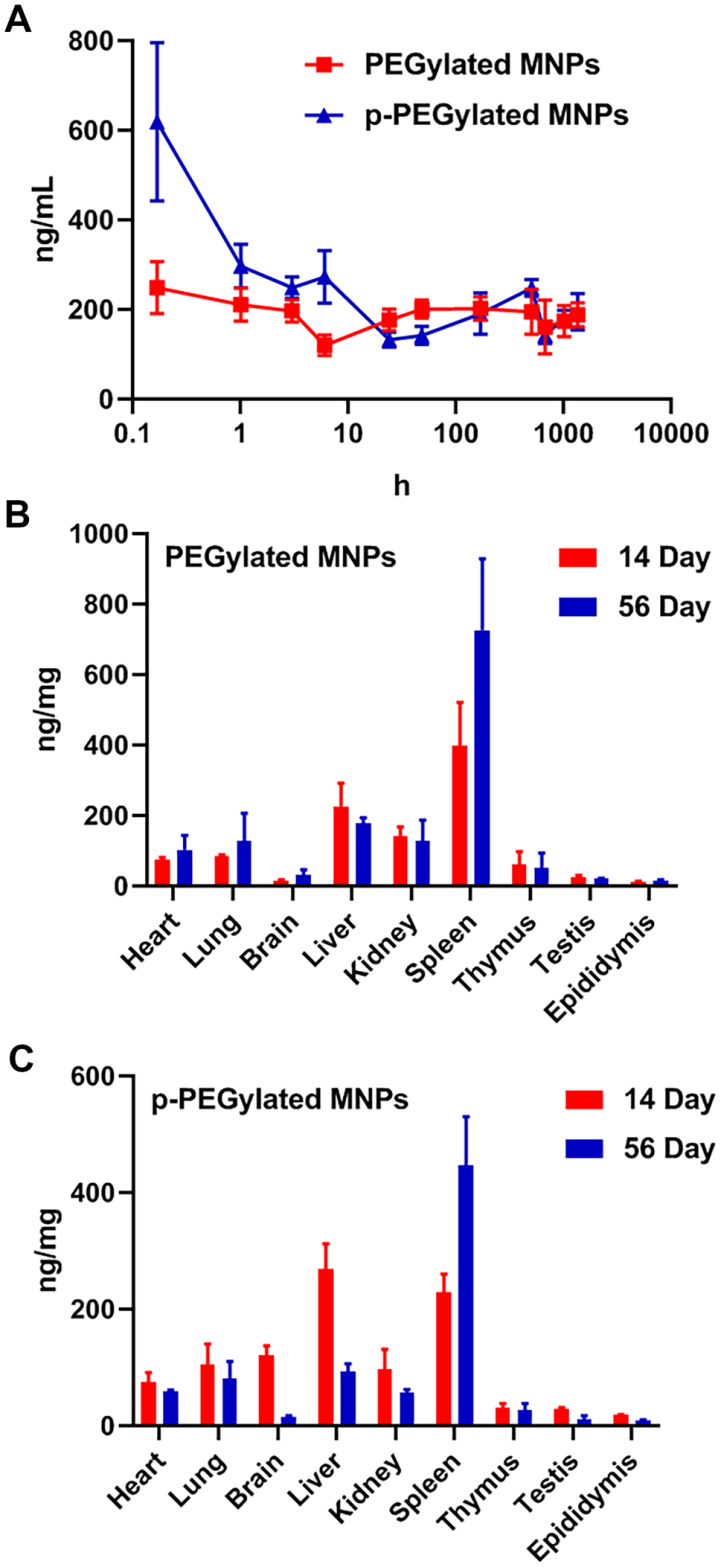
Biodistribution of PEGylated MNPs and p- PEGylated MNPs. (**A**) Content of PEGylated MNPs and p- PEGylated MNPs in the plasma from 5 min to 56 days post-administration. (**B**) Content of PEGylated MNPs in major organs of rats at 14 days and 56 days post-administration. (**C**) Content of p-PEGylated MNPs in major organs of rats at 14 days and 56 days post-administration.

The biodistribution of nanoparticles in various tissues were demonstrated in Fig. 3. The content of PEGylated MNPs in the plasma was about 200 ng/mL at 5 min post-administration and maintained at this level till 56 days post-administration. For animals dosed with the PEGylated MNPs, spleen, liver and kidneys were found as the major targeted organs, while few particles were also found in brain, testis, and epididymis. In contrast, the content of p-PEGylated MNPs in the plasma reached about 600 ng/mL at 5 min post-administration, and it was rapidly dropped to about 300 ng/mL from 1 to 8 h post-administration and stayed around 200 ng/mL till 56 days. The spleen, liver, and kidneys were the organs with the most p-PEGylated MNPs accumulated, which was similar to the PEGylated MNPs. Comparing with the PEGylated MNPs, however, more p-PEGylated MNPs were found in the liver and the brain of the rats on 14 days post-administration.

### Overall toxicity

**Figure 4:**
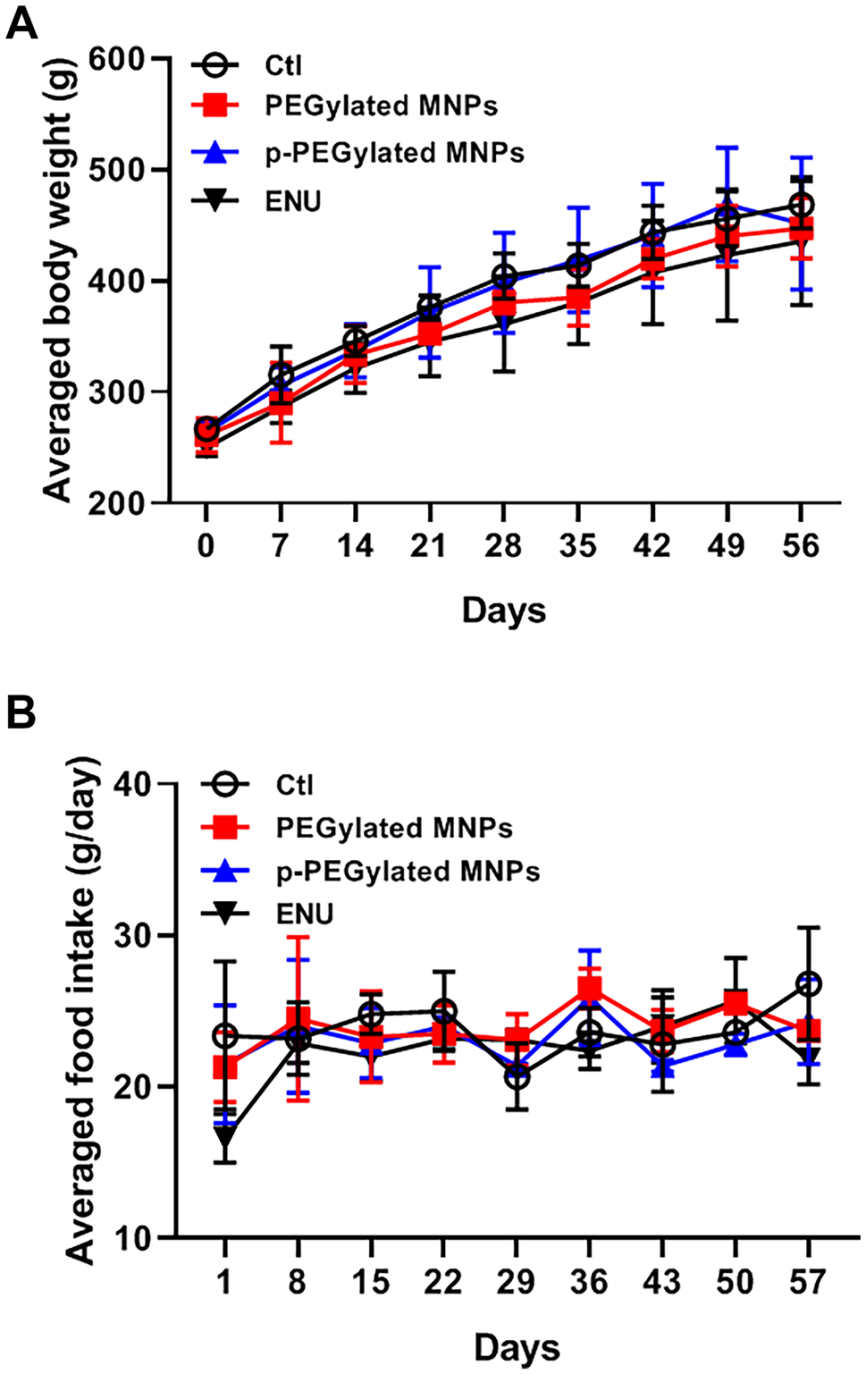
Averaged body weights and food intakes. Body weights (**A**) and food intakes (**B**) of animals from different groups are measured every week and the averaged data are summarized.

There was no obvious abnormal symptom observed in all rats up to 56 days post-administration of PEGylated MNPs and p-PEGylated MNPs. The averaged body mass and food intake post-administration were summarized in Fig. 4A,B, respectively, and no significant change was observed among all groups.

**Figure 5:**
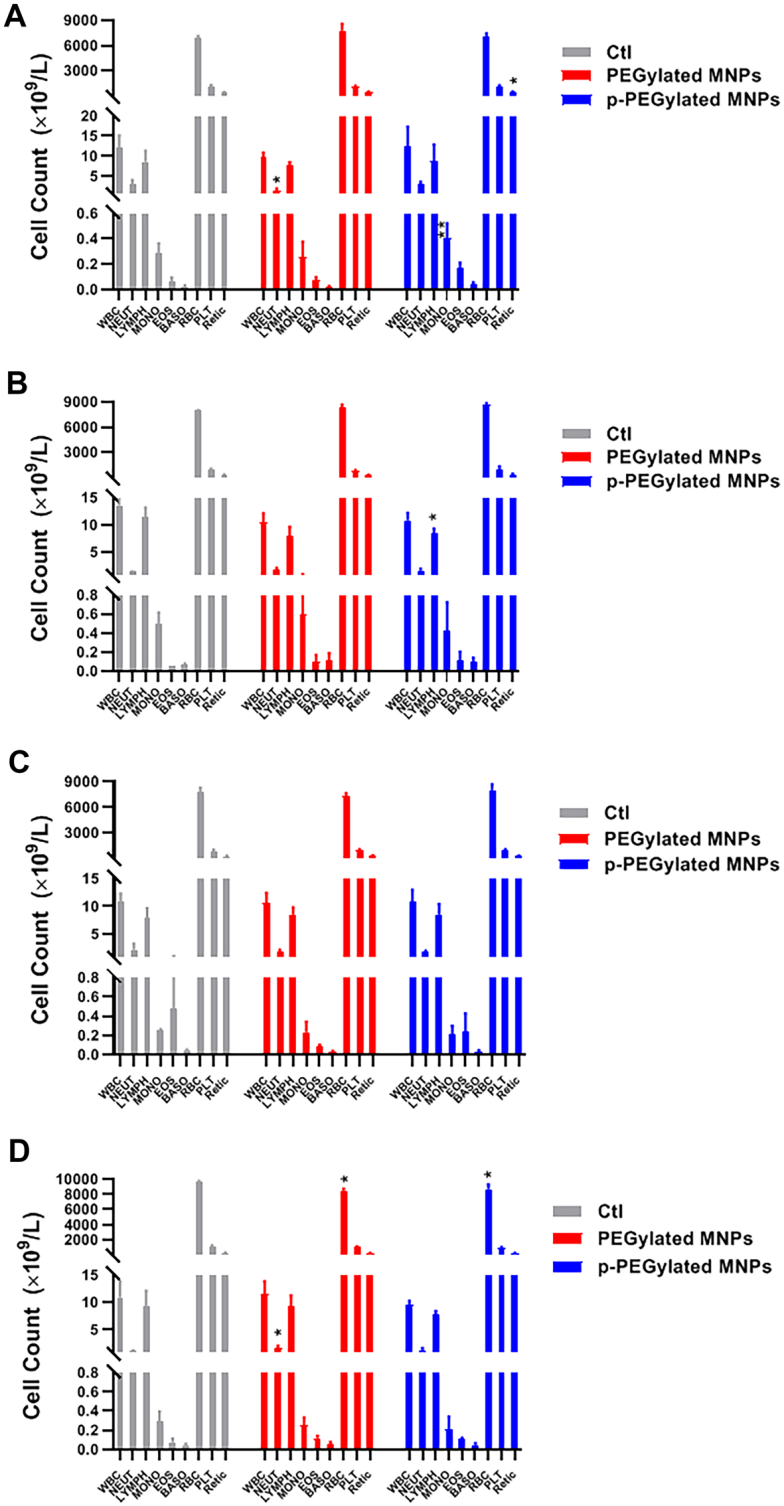
Changes on hematological examination indexes. Leukocyte (WBC), neutrophil (NEUT), lymphocyte (LYMPH), monocyte (MONO), Eosnophil (EOS), basophil (BASO), erythrocyte (RBC), platelet (PLT) and reticulocyte (Retic) in blood samples from animals of different groups on 7 days (**A**), 14 days (**B**), 28 days (**C**) and 56 days (**D**) after administration were counted and summarized respectively. One-way ANOVA, comparing with the Control (Ctl), *p < 0.05, **p < 0.01.

The hematological indexes of animals administrated with PEGylated MNPs and p-PEGylated MNPs were summarized in Fig. 5. For the animals administrated with PEGylated MNPs, the averaged neutrophil count in was significantly lower than that of the Control group (*p* < 0.01) on 7 days post-administration, while the averaged neutrophil count was significantly higher than that of the Control group (*p* < 0.05) on 56 days post-administration. In addition, the averaged RBC count was significantly lower than that of the Control group (*p* < 0.05) on 56 days post-administration. For the animals administrated with p-PEGylated MNPs, the averaged monocyte count was higher than that of the Control group (*p* < 0.01) on 7 days after dosing, while the averaged lymphocyte count was lower than that of the Control group (*p* < 0.05) on 14 days post-administration. The averaged Retic count in animals administrated with p-PEGylated MNPs was significantly higher than that of the Control group on 7 days post-administration, while the averaged RBC count was significantly lower than that of the Control group on 56 days post-administration.

**Figure 6:**
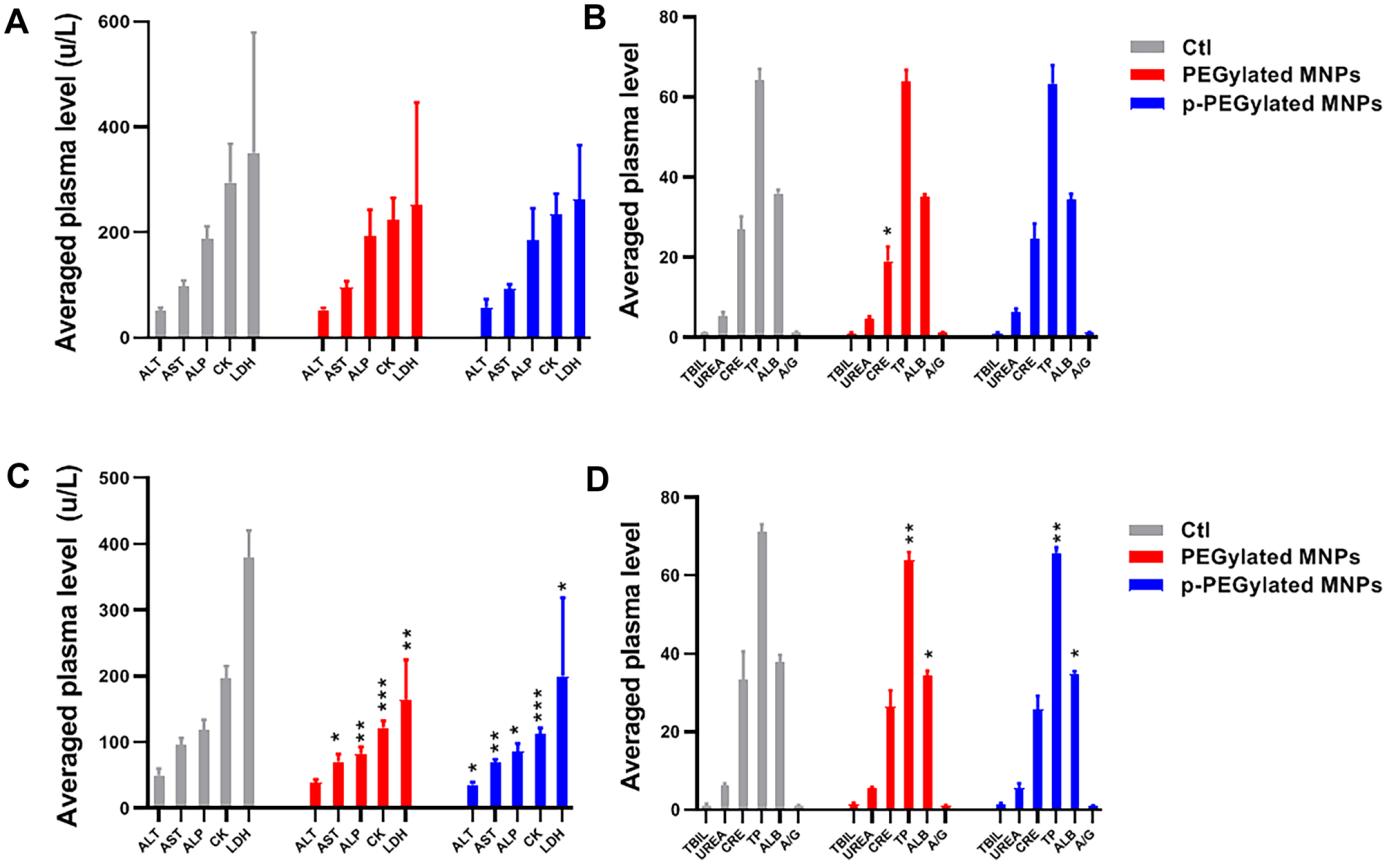
Changes on serum biochemical examination indexes. Alanine transaminase (ALT, µ/L), aspartate aminotransferase (AST, μ/L), alkaline phosphatase  (ALP, μ/L), creatine kinase (CK. μ/L), lactate dehydrogenase (LDH, μ/L), total bilirubin (TBIL,μmol/L), urea nitrogen (UREA,mmol/L), creatinine (CRE,μmol/L), total protein (TP,g/L), albumin (ALB,g/L), ratio of albumin to globulin (A/G) in blood samples from animals of different groups on 14 days (**A**, **B**), and 56 days (**C**, **D**) post-administration were counted and summarized respectively. One-way ANOVA, comparing with the Control (Ctl), *p < 0.05, ** p < 0.01, ***p < 0.001.

Biochemical examination data of animals were summarized in Fig. 6. For the animals administrated with PEGylated MNPs, the averaged CRE was significantly lower than that of the Control group (*p* < 0.05) on 14 days post-administration. The averaged AST, ALP, CK, LDH, TP and ALB in animals dosed with PEGylated MNPs were significantly lower than that of the Control group (*p* < 0.05, *p* < 0.01, *p* < 0.001) on 56 days post-administration. Similar pattern was also observed in animals dosed with p-PEGylated MNPs, as the averaged ALT, AST, ALP, CK, LDH, TP and ALB were significantly lower than that of the Control group (*p* < 0.05, *p* < 0.01, *p* < 0.001) as well.

### Pathological examination

**Figure 7:**
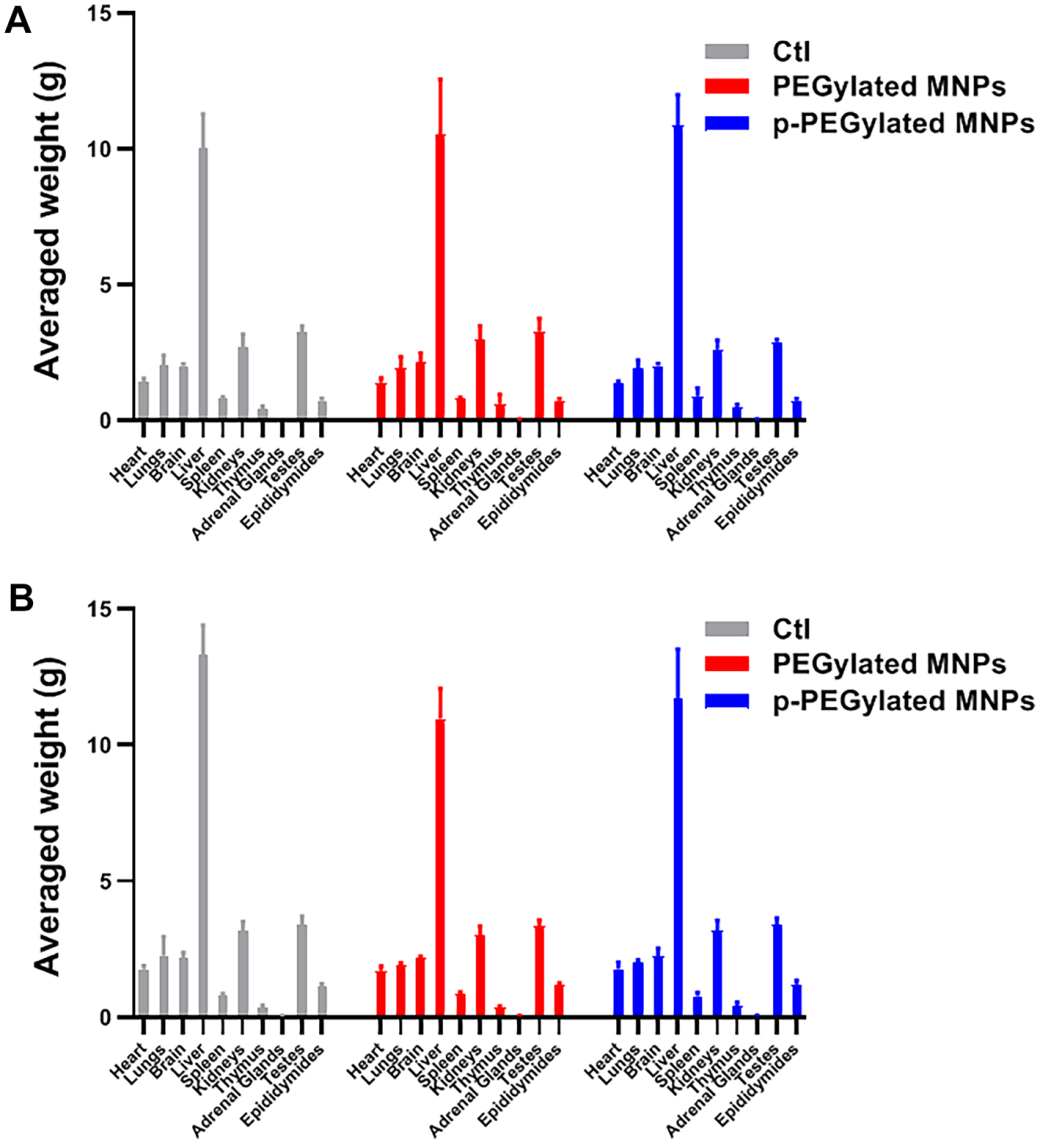
Changes on organ weights. Weights of heart, lungs, brain, liver, spleen, kidneys, thymus, adrenal glands, testes and epididymides of animals from different groups on 14 days (**A**) and 56 days (**B**) after administration were measured and summarized.

**Figure 8:**
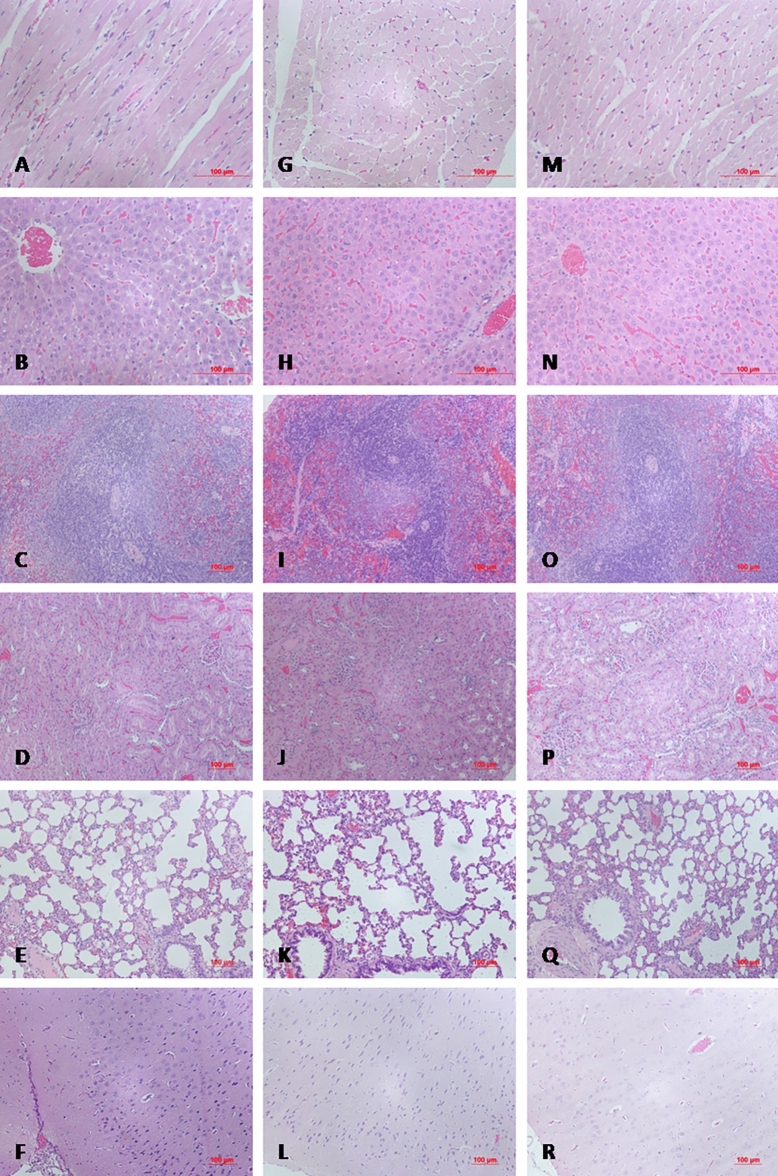
Pathological Changes. Compared with the control group (**A**–**F** **A**,**B** 200 × **C**–**F** 100 ×), there were no obvious histopathological changes associated with administration of PEGylated MNPs and p-PEGylated MNPs in the heart, liver, spleen, kidney, lung, and brain of PEGylated MNPs group (**G**–**L** **G**,**H** 200 × **I**–**L** 100 ×) and p-PEGylated MNPs group (**M**–**R** **M**,**N** 200 × **O**–**R** 100 ×),respectively.

The averaged organ weights of animals on 14 days and 56 days after dosing were summarized in Fig. 7. There was no statistical difference found in organ weights among groups. Compared with control group, there were no obvious histopathological changes associated with administration of PEGylated MNPs and p-PEGylated MNPs in the heart, liver, spleen, kidneys, lungs, and brain of PEGylated MNPs group and p-PEGylated MNPs group (Fig. 8). The other histopathological changes were spontaneous and incidental, which were not related to the administration of PEGylated MNPs and p-PEGylated MNPs.

### Immunotoxicity

**Figure 9:**
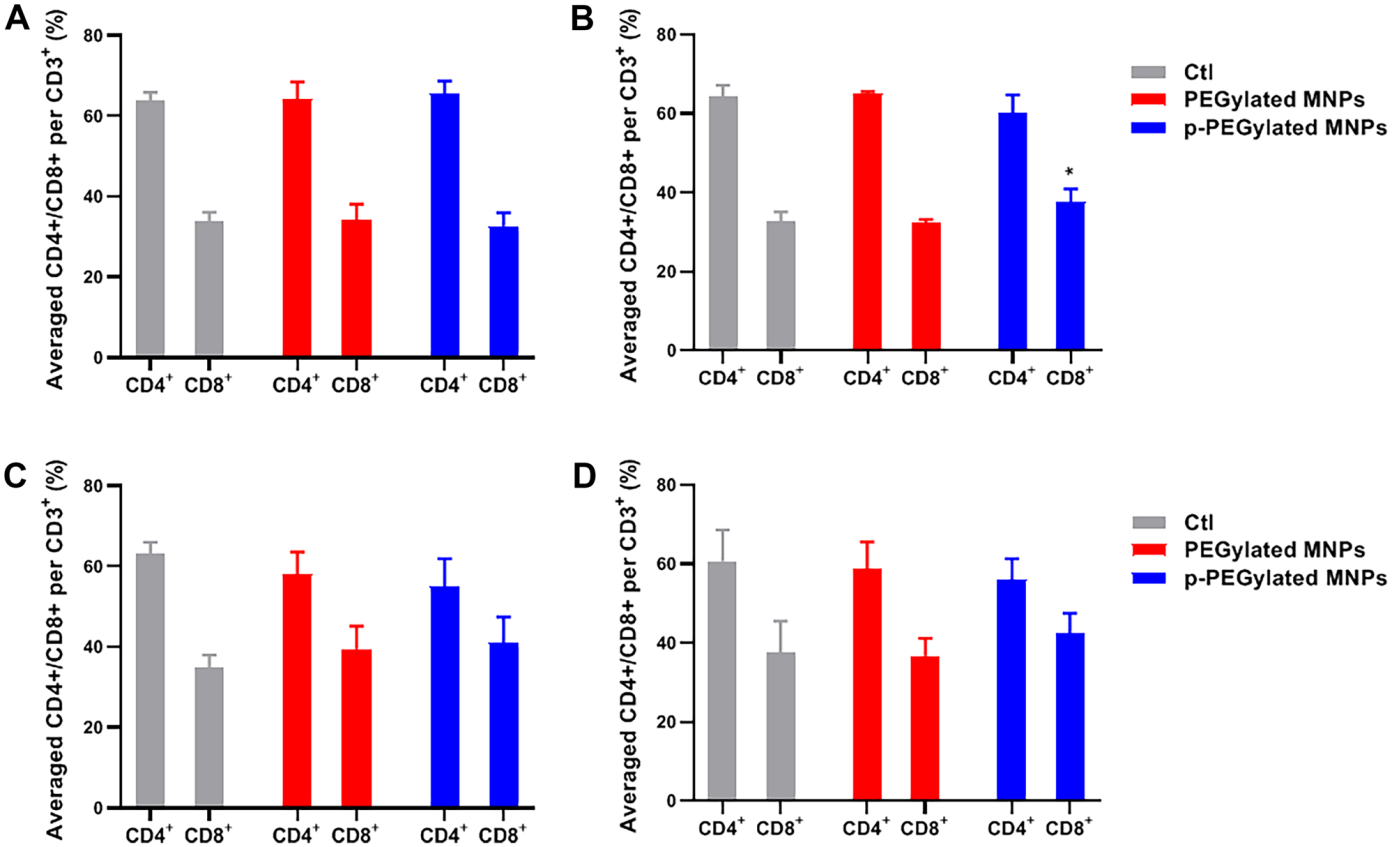
Changes on CD4^+^ and CD8^+^ T lymphocytes. CD4^+^ and CD8^+^ lymphocytes in total 20,000 T cells (CD3^+^positive) from rats blood samples were recognized and counted by flow cytometry on 7 days (**A**), 14 days (**B**), 28 days (**C**) and 56 days (**D**) post-administration, and the data were summarized. One-way ANOVA, comparing with the Control (Ctl), *p < 0.05.

The averaged expression levels of CD4^+^ and CD8^+^ in animals administered with PEGylated MNPs and p-PEGylated MNPs were summarized in Fig. 9. The averaged CD8^+^ T lymphocytes were significantly higher than that of the Control group (*p* < 0.05) on 14 days post-administration. CD8^+^ T lymphocytes are known as killer T cells, which are able to attack the exogenous granules, and recruit cytokines for activating the immune responses.

### Cytokine profiles

**Figure 10:**
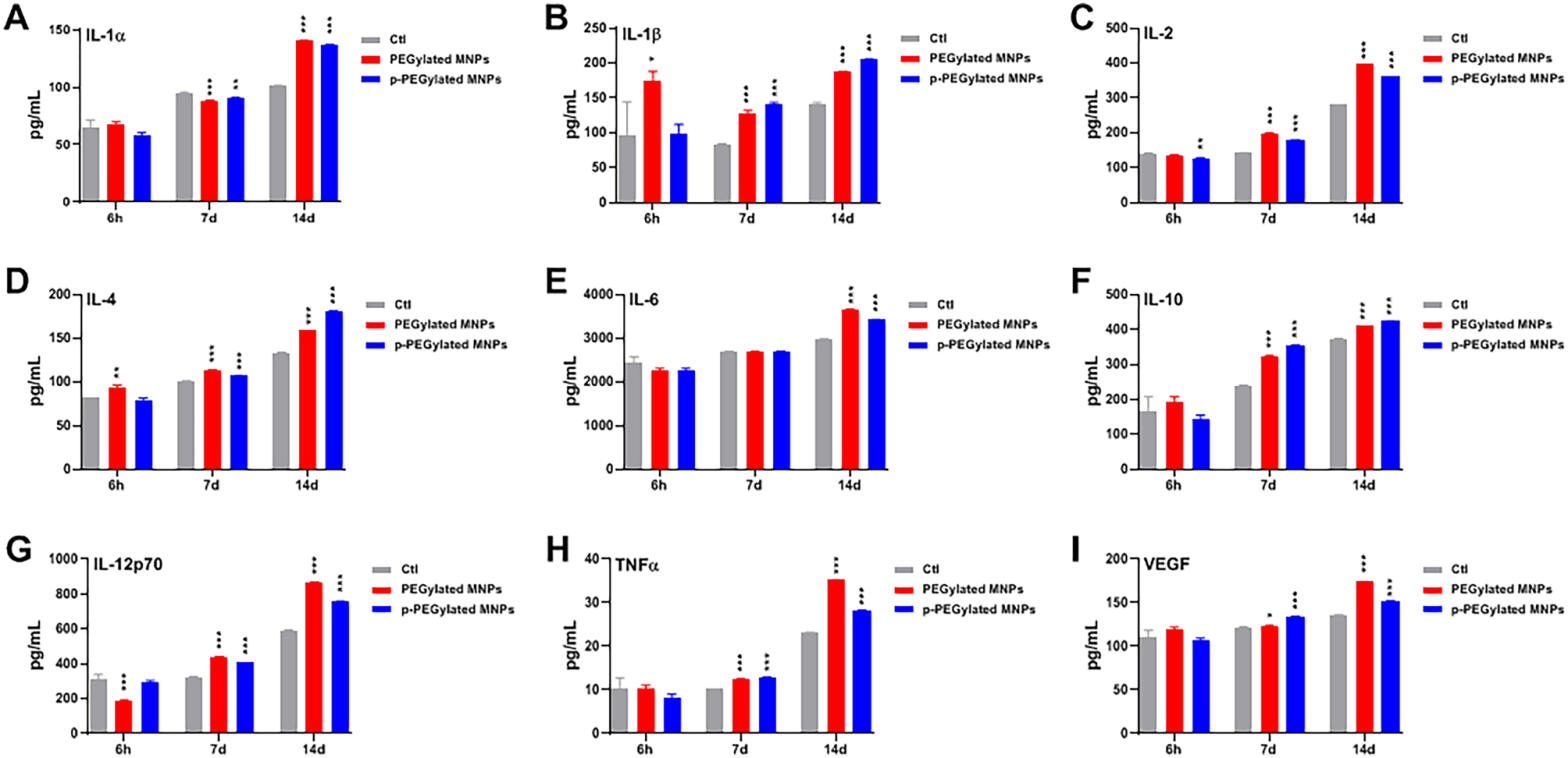
Changes on serum cytokine profile in rats. The levels of serum IL-1α (**A**), IL-1β (**B**), IL-2 (**C**), IL-4 (**D**), IL-6 (**E**), IL-10 (**F**), IL-12p70 (**G**), TNF-α (**H**) and VEGF (I) in rats at 6 h, 7 days and 14 days post administered PEGylated MNPs and p-PEGylated MNPs were summarized. One-way ANOVA, comparing with the Control (Ctl),*p < 0.05, **p < 0.01, ***p < 0.001.

Cytokines are a class of small molecular proteins with wide biological activity synthesized and secreted by immune cells and some non-immune cells after stimulation. The cytokine expression levels reflect the systemic immune response. As nanomaterials could trigger cellular damage and inflammatory responses through phagocytes and dendritic cells in the immune system, hence, cytokine expression detection was included to evaluate the immunotoxicity. The inflammation potentials of PEGylated MNPs and p-PEGylated MNPs were evaluated by detecting the levels of cytokines released in the serum (Fig. 10). For animals administered PEGylated MNPs, the levels of IL-1β, IL-4 were significantly higher than that of the Control group at 6 h, 7 days and 14 days post-administration, the levels of IL-2, IL-10, IL-12p70, TNFα and VEGF were significantly higher than that of the Control group at 7 days and 14 days post-administration, and the level of IL-1α and IL-6 were significantly higher than that of the Control group at 14 days post-administration. In addition, the level of IL-1α was lower than that of the Control group at 7 days post-administration, and the level of IL-12p70 was lower than that of the Control group at 6 h post-administration. For animals administered p-PEGylated MNPs, the level of IL-2 was significantly higher than that of the Control group at 7 days and 14 days post-administration, the levels of IL-1β, IL-2, IL-4, IL-10, IL-12p70, TNFα and VEGF were significantly higher than that of the Control group at 7 days and 14 days post-administration, and the level of IL-1α and IL-6 was significantly higher than that of the Control group at 14 days post-administration. The level of IL-1α was lower than that of the Control group at 6 h post-administration, and the level of IL-2 was lower than that of the Control group at 6 h post-administration. The variation trends between the two MNPs are essentially similar, except that the IL-1β and IL-4 were more readily to be up-regulated by the PEGylated MNPs. Together, all the cytokines could be elevated by the administration of MNPs within 14 days, and a time-dependent trend was observed.

### Genotoxicity

**Figure 11:**
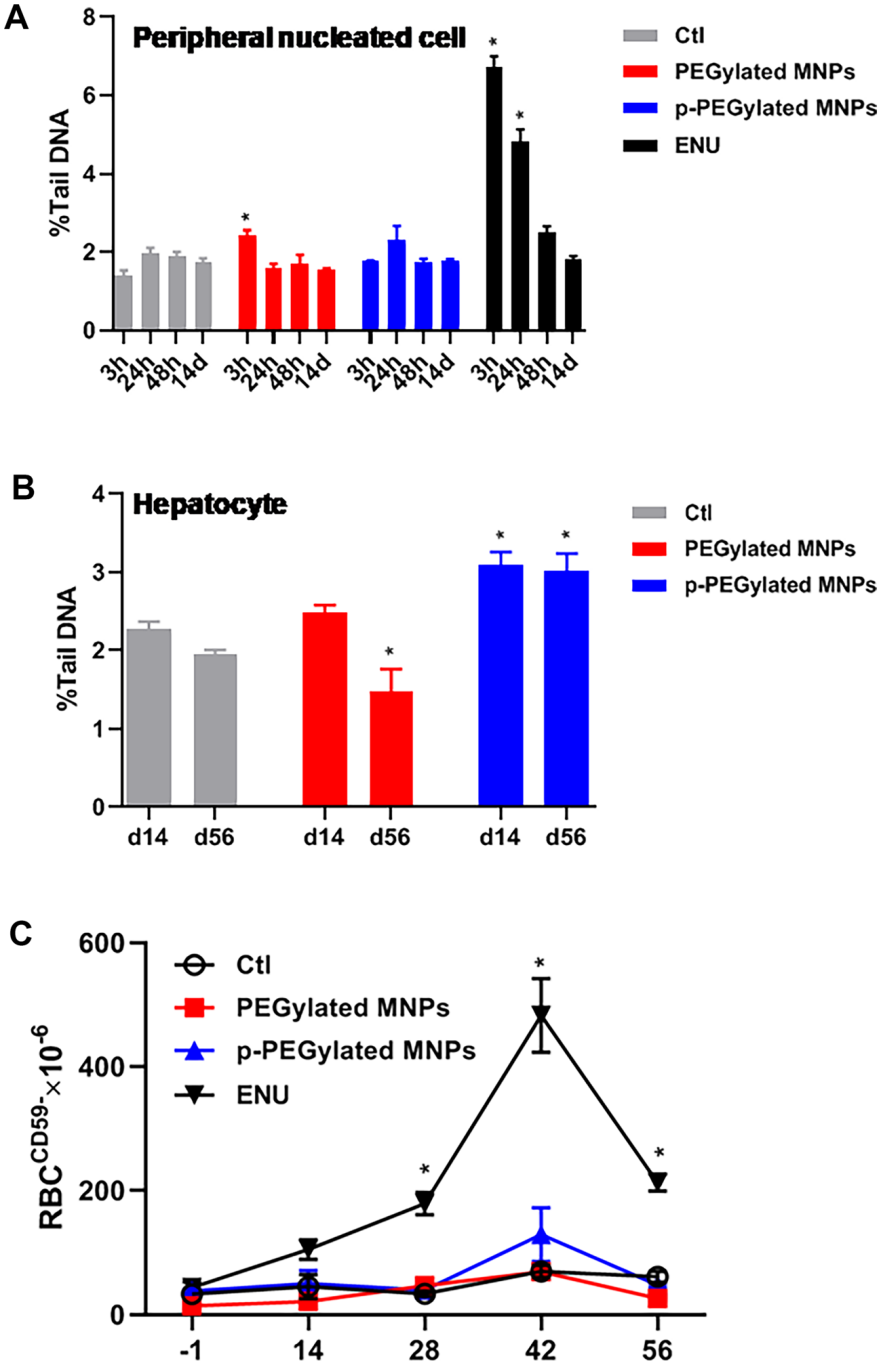
Genotoxicity potential of PEGylated MNPs and p-PEGylated MNPs. Comet assay were performed using peripheral nucleated cells and hepatocytes to investigate the DNA damage potential of PEGylated MNPs and p-PEGylated MNPs, and %tail DNA were evaluated to determine the extents of DNA breakage in blood samples(**A**) and hepatocytes(**B**) at different time points. In addition, *Pig-a* gene assay was performed to evaluate the gene mutation risk and the averaged RBC^CD59-^ was counted using flow cytometry (**C**). ENU group was included as positive control. One-way ANOVA, comparing with the Control (Ctl),*p < 0.05.

The genotoxicity potential is related to the carcinogenic risk of the nanomaterials, which has become a concern before applied to human. The DNA breakage potentials of peripheral nucleated cells and hepatocytes in animals administrated with PEGylated MNPs and p-PEGylated MNPs were evaluated by Comet assay (single cell gel electrophoresis assay, Fig. 11A,B). Comet assay could detect the DNA breakage potential on the targeted and accumulation tissue, and it is a sensitive indicator for detecting the DNA damage induced within a short period. It is found that the averaged %Tail DNA of peripheral nucleated cells in animals dosed with PEGylated MNPs for 3 h was significantly higher than that of the Control group (*p* < 0.05), while no difference was observed at 24 h, 48 h, and 14 days post-administration in those dosed with PEGylated MNPs, suggesting this damage is transient and repairable. However, for those administrated with p-PEGylated MNPs, the averaged %Tail DNA of hepatocytes on 56 days post-administration was significantly higher than that of the Control group (*p* < 0.05), however, which was not observed in animals dosed with PEGylated MNPs, indicating the p-PEGylated MNPs are readily persist in the liver and introduce DNA damage than PEGylated MNPs.

The in vivo gene mutation risk of test article could be detected by recognizing the CD59- expression condition on the surface of rat RBC or RET. The gene mutation risk was evaluated by determine the incidence of RBC^CD59-^ in the peripheral blood. Our data (Fig. 11C) demonstrated that both PEGylated MNPs and p-PEGylated MNPs were not mutagenic as no significant change on the rat RBC^CD59-^was observed.

## Discussion

The physical and chemical properties of MNPs pose a unique biological mode of action, as well as a certain toxicity risk. Tissue distribution, immunotoxicity and potential genotoxicity caused by a long-term persistence are the main focuses among the toxicity risks of nanomaterials^21^. In this study, two representative MNPs (PEGylated and p-PEGylated MNPs) were single administered in SD rats and toxicity endpoints were continuously detected for 56 days post-administration. Our results demonstrated that both nanoparticles could be rapidly cleared from plasma and enter tissues, such as, liver, kidneys and spleen. Although MNPs showed accumulation trends multiple organs (for instance, PEGylated MNPs could accumulated in the heart, lung, brain and spleen, p-PEGylated MNPs could accumulated in the spleen) of rats with prolonged observation time, no related pathological changes were found. In addition, PEGylated MNPs are more likely to up-regulate the expression levels of Th2 type cytokines and trigger inflammatory pathways.

Bio-distribution serves as the basis for the interpretation of safety data, allows us to identify the main toxic target organs, and provide important information on the toxicological risks. We have demonstrated that PEG (poly(ethylene glycol))-coated MNP could accumulated in blood, liver and tumor within 72 h. In this study, the distribution characteristics of PEGylated and p-PEGylated MNPs were observed till 56 days post administration^6^. This study revealed that both MNPs were rapidly cleared from the circulatory system subsequent to intravenous injection, while they maintained for up to 56 days at a level around 200 ng/mL in the plasma. The distribution characteristics of MNPs were similar at 14 days and 56 days post-administration, and were predominantly accumulated in spleen, liver, kidneys, lungs, brain, and heart. The overall content of PEGylated MNPs in the tissue was higher than that of p-PEGylated. The contents of PEGylated MNPs in heart, lungs and spleen at 56 days post-administration were higher than that of the 14 days post administration. The content of p-PEGylated MNPs at 56 days post administration in spleen was higher than that of 14 days post administration, while the contents of p-PEGylated MNPs in other tissues, especially liver, were lower than that of 14 days post administration. These suggested that PEGylated MNPs are more likely to be accumulated in the spleen, liver, and kidneys, comparing with the p-PEGylated MNPs. In consistent with previous studies^22–24^, the physicochemical characteristics of nanoparticles such as size, surface morphology and surface charge, could influence their composition and quantity of associated proteins, resulted in different toxicities and in vivo behaviors. For inorganic nanoparticles, even with the same core size and chemical composition, may also present quite different biological behaviors due to the differences in surface properties dependent on the interactions between the nanoparticles and plasma proteins, leading to different fates and metabolic pathways.

PEG is currently the most used modification for nanomaterials for therapeutic purpose^25^. PEG is often used for particle coating to increase hydrophilicity and prolong particle circulation time. Both p-PEGylated MNPs and PEGylated MNPs have long half-lives and good biocompatibility in the body, promising long-term tracking possibility. Surface charge of the MNPs is largely attributed to the coating materials. According to previous findings, the negatively charged MNPs demonstrated greater uptake by the resident macrophages than their neutral counterparts ^26^^.^ The PEGylated MNPs and p-PEGylated MNPs used in this study exhibited similar biodistribution. This may resulted from their similar DLS (63.7 nm and 41.8 nm in water), and positive or neutral electrical property, respectively. In addition, PEG has been excelled in prolonging the circulation of nanoparticles, while evidence shown that the α-PEG antibodies elicited by PEGylated system (predominantly IgM response) could be responsible for the accelerated blood clearance ^27–29^. The observed difference might due to the variations on the production of anti-PEG antibodies. At present, MNPs are mainly applied in the diagnosis and treatment of liver tumor, and the distribution characteristics are consistent with its clinical needs. In addition, spleen is the main component of reticuloendothelial system; these results also indicated the potential immunotoxicity and hepatotoxicity of MNPs. This study provided evidence for PEGylated/p-PEGylated MNPs in the therapy of brain disease, as they could cross the blood–brain barrier. In addition, the enrichment of PEGylated/p-PEGylated MNPs in spleen, suggested their application prospects in splenic imaging. For nanoparticles with such long internal circulation time, they are suitable for labeling tumor targeted antibodies and long-term tracking of tumors.

Immunotoxicity of nanomaterials has arouse a wide attention, and the charge and hydrophobic interactions of MNPs could affect the interaction between nanoparticles and immune proteins ^30^. The current understandings on the effect of MNPs on T-cell function or mechanisms of immunotoxicity are incomplete. Several earlier studies attempted to investigate how MNPs influence T-cell function and found no profound effects. Our data suggested that the number of CD8^+^T lymphocytes in animals administered p-PEGylated MNPs increased, indicating that p-PEGylated MNPs could activate the immunotoxic response in vivo. This may also associate with the longer circulation time of p-PEGylated MNPs. In addition, both MNPs could up-regulate various cytokines such as IL-1α, IL-1β, IL-2, IL-4, IL-10, IL-12p70, TNFα and VEGF. Inflammation is an important toxic mechanism mediated by nanoparticles. As resulted, PEGylated MNPs are more likely to up-regulate the expression levels of Th2 type cytokines IL-1β and IL-4.It has been reported that the MNPs could polarize the balance between Th1 and Th2 type cytokines^31^. Moreover, IL-1β is considered as a key pro-inflammatory cytokine and is associated with the inflammatory effects of many nanoparticles, for instance, the silver nanoparticles^32^ and double-walled carbon nanotubes^33^. Previous study ^34^ suggested that monophosphate modified nanoparticles could result in low level of immunotoxicity and coordinate a mild inflammatory response in macrophages, as they are recognized by TLR receptors in immune cells, activate the NF-κB pathway, and induce pro-inflammatory cytokines, including IL-1β and TNF-α, ultimately leading to multiple cellular inflammatory responses including cytokine secretion.

The effects of MNPs on T cells in vivo depend on many factors, including nanoparticle physicochemical properties, dose, and the route of administration. Zhu et al. demonstrated Th1 polarization and exaggeration of DTH reaction to oval bumin in both OVA-sensitized and unchallenged BALB/c mice, indicating the immunostimulatory effect of magnetic MNPs^35^. Previous study suggested that Ferumoxide activated a Th1-type immune response and inflammation, where iron oxide accumulation in the lungs was associated with elevated levels of Th1 cytokines and chemokines in the bronchoalveolar lavage ^35,36^. Several studies have shown induction of a variety of cytokines/chemokines in response to MNPs treatment. However, the available data are controversial, and comparison between the studies is challenging due to differences in the immune-cell types, particle physicochemical properties, concentrations, doses, routes of administration, and time employed. More studies and better experimental models are needed to be explored.

The in vitro genotoxicity potentials of diverse nanomaterials have been extensively studied, while it is for the first time reported the genotoxicity risk of MNPs after a long term (2 month) presence in the circulation. We demonstrated that both MNPs were not mutagenic in vivo, by presenting that the frequency of mutated *Pig-a* gene in the peripheral blood sample were not increased 56 days post the exposure of MNPs. In addition to the bio-distribution data, it is suggested that single exposure of PEGylated MNPs and p-PEGylated MNPs at a low plasma level (200 ng/mL) would not introduce gene mutation in vivo. However, PEGylated MNPs induced a mild DNA breakage on th peripheral lymphocytes at 3 h post-administration, while there was no obvious DNA breakage at 24 h post-administration. This might be related to the high contents of PEGylated MNPs in the circulation immediately after the intravenous injection. As PEGylated MNPs were rapidly cleared in peripheral blood, the above DNA damage was not sustained and repairable. At 56 days post-administration, p-PEGylated MNPs resulted in DNA damage in hepatocytes, which may be associated with its higher content and persistent effect in the liver at this time. However, due to the limitation of the study design, it is pity that the dose–response trend was not able to observe. As no gene mutation potential was observed, the mild DNA damage might not be fixed to a heritable mutation. Comet assay could sensitively detect the DNA damage that caused by MNPs^37^, and the underlying mechanism is related to the oxidative stress. After absorption by the cell, the iron oxide nanoparticles (IONPs) could locate in the acidic medium of the lysosome, where it is metabolized and produces free iron ions into the cell. As reviewed by Mai et al^38^, it is proposed that the MNPs could produce reactive oxygen species (ROS) and enhance the formation of intracellular ROS, which play a major role and results in cell damage and death, via Fenton and Haber–Weiss reaction. In specific, the iron ions released by IONPs promote the Fenton reaction to produce ROS from H_2_O_2_ and superoxides, and subsequently lead to oxidative stress and ultimately cell death^39,40^. It was also implied that the toxicity produced by ROS could be suppressed, when an appropriate design is available. This feature of MNPs might have great application prospect in the fields of alternating magnetic fields, radiation, and chemotherapy. Overall, the DNA damage tendency implied the oxidative stress induced by MNPs, and this phenomenon might be absent when lower doses are administered. On account of the rapid clearance of MNPs in the blood, as well as the lack of data from multiple dose-groups, the genotoxic risk of MNPs could be further investigated.

This study revealed the bio-distribution of two kinds of well prospective PEGylated MNPs and p-PEGylated MNPs, allows us to identify the main toxic target organs, and provide important information on the toxicological risks. Some differences about the immunotoxicity are observed. Differences in immune responses (e.g. Th1-biased vs Th2-biased animals) could be considered, and further mechanism studies on the immune reactions in response to the MNP formulations are still needed. For a follow-up study, it is recommended to perform the tissue distribution and toxicity study in at least two species for comparison, and special attention should be paid on metabolic differences between the species. In addition, three dose groups should be set up in toxicity study with a prolonged observation period, considering the delayed toxicity risk.

## Conclusion

The MNPs could be used in targeted cancer imaging and drug delivery. The PEGylated MNPs and p-PEGylated MNPs used in this study have exhibited outstanding performance as MRI contrast agents. PEGylated surface modification greatly improved biocompatibility. Though the extensive efforts on the safety assessment of iron oxidative nanoparticles have been made^15^, standardized guidelines on the risk evaluation of nanoparticles have not been formulated. Due to the long-term accumulation of nanomaterials in targeted tissues, the observation period should be extended to at least 4 to 6 weeks post-administration for a safety risk assessment. It is also preferable to perform toxicokinetic study/bio-distribution study, immunotoxicity, and central neurotoxicity along with the toxicity study. Nevertheless, cytokines and inflammatory response shall be focused as indicators for understanding the potential toxic effects. Besides, central nerve system (CNS) toxicity is one of the main toxicity concern of MNPs. IONPs has been reported as carriers for delivering therapeutic molecules in the CNS, while they could introduce neural tissue injury via mechanisms, such as oxidative stress and free iron accumulation^41^. In this study, we revealed that both p-PEGylated MNPs and PEGylated MNPs have long half-lives in the body, promising long term tracking possibility. In addition, p-PEGylated MNPs are less prone to be accumulated in the CNS and many other tissues, indicating a lower toxicity risk. This study also demonstrated a research approach for the non-clinical safety evaluation of nanoparticles. It also provided comprehensive valuable safety data for PEGylated and p-PEGylated MNPs, for promoting the clinical application and bio-medical translation of such MNPs with PEG modifications in the cancer diagnosis and therapy.

## Data Availability

The data used to support the findings of this study are included within the article.

## References

[1] Weissleder R, Pittet MJ (2008). Imaging in the era of molecular oncology. Nature.

[2] Lee JH, Huh YM, Jun YW (2007). Artificially engineered magnetic nanoparticles for ultra-sensitive molecular imaging. Nat. Med..

[3] Salunkhe AB, Khot VM, Pawar SH (2014). Magnetic hyperthermia with magnetic nanoparticles: A status review. Curr. Top. Med. Chem..

[4] Weinstein JS, Varallyay CG, Dosa E (2010). Superparamagnetic iron oxide nanoparticles: Diagnostic magnetic resonance imaging and potential therapeutic applications in neurooncology and central nervous system inflammatory pathologies, a review. J. Cereb. Blood Flow Metab..

[5] Gómez-Vallejo V, Puigivila M, Plaza-García S (2018). PEG-copolymer-coated iron oxide nanoparticles that avoid the reticuloendothelial system and act as kidney MRI contrast agents. Nanoscale.

[6] Liu S, Jia B, Qiao R (2009). A novel type of dual-modality molecular probe for MR and nuclear imaging of tumor: Preparation, characterization and in vivo application. Mol Pharm..

[7] Zanganeh S, Hutter G, Spitler R (2016). Iron oxide nanoparticles inhibit tumour growth by inducing pro-inflammatory macrophage polarization in tumour tissues. Nat. Nanotechnol..

[8] Zhu MT, Wang Y, Feng WY (2010). Oxidative stress and apoptosis induced by iron oxide nanoparticles in cultured human umbilical endothelial cells. J. NanosciNanotechnol..

[9] Canivet L, Denayer FO, Dubot P, Garçon G, Lo Guidice JM (2021). Toxicity of iron nanoparticles towards primary cultures of human bronchial epithelial cells. J. ApplToxicol..

[10] Zhu MT, Feng WY, Wang Y (2009). Particokinetics and extrapulmonary translocation of intratracheally instilled ferric oxide nanoparticles in rats and the potential health risk assessment. Toxicol. Sci..

[11] Salimi M, Sarkar S, Fathi S (2018). Biodistribution, pharmacokinetics, and toxicity of dendrimer-coated iron oxide nanoparticles in BALB/c mice. Int. J. Nanomed..

[12] Li L, Ishdorj G, Gibson SB (2012). Reactive oxygen species regulation of autophagy in cancer: Implications for cancer treatment. Free RadicBiol. Med..

[13] Gao Z, Ma T, Zhao E (2016). Small is smarter: Nano MRI contrast agents - Advantages and recent achievements. Small..

[14] Di Gioacchino M, Petrarca C, Lazzarin F (2011). Immunotoxicity of nanoparticles. Int. J. ImmunopatholPharmacol..

[15] Shah A, Dobrovolskaia MA (2018). Immunological effects of iron oxide nanoparticles and iron-based complex drug formulations: Therapeutic benefits, toxicity, mechanistic insights, and translational considerations. Nanomedicine.

[16] Ansari MO, Parveen N, Ahmad MF (2019). Evaluation of DNA interaction, genotoxicity and oxidative stress induced by iron oxide nanoparticles both in vitro and in vivo: Attenuation by thymoquinone. Sci. Rep..

[17] Li Z, Wei L, Gao MY, Lei H (2005). One-pot reaction to synthesize biocompatible magnetite nanoparticles. AdVMater.

[18] Jia Q, Zeng J, Qiao R (2011). Gelification: An effective measure for achieving differently sized biocompatible Fe_3_O_4_ nanocrystals through a single preparation recipe. J. Am. Chem. Soc..

[19] Zeng J, Jing L, Hou Y (2014). Anchoring group effects of surface ligands on magnetic properties of Fe_3_O_4_ nanoparticles: Towards high performance MRI contrast agents. Adv. Mater..

[20] Wang D, Dan M, Ji Y, Wu X, Xu L, Wen H (2018). Single-dosed genotoxicity study of gold nanorod core/silver shell nanostructures by Pig-a, micronucleus, and comet assays. J. Biomed. Nanotechnol..

[21] Sekhon BS, Kamboj SR (2010). Inorganic nanomedicine–part 2. Nanomedicine.

[22] Lu J, Chen Y, Ding M (2022). A 4arm-PEG macromolecule crosslinked chitosan hydrogels as antibacterial wound dressing. Carbohydrate Polym..

[23] Wang Y, Zhai W, Cheng S (2023). Surface-functionalized design of blood-contacting biomaterials for preventing coagulation and promoting hemostasis. Friction.

[24] Fereshteh D, Masoud TM (2018). Magnetic delivery of antitumor carboplatin by using PEGylated-Niosomes. DARU J. Pharma. Sci..

[25] Jokerst JV, Lobovkina T, Zare RN, Gambhir SS (2011). Nanoparticle PEGylation for imaging and therapy. Nanomedicine.

[26] Maurizi L, Papa AL, Dumont L, BouyerF WP, Vandroux D (2015). Influence of surface charge and polymer coating on internalization and biodistribution of polyethylene glycol-modified iron oxide nanoparticles. J. Biomed. Nanotechnol..

[27] Laverman P, Carstens MG, Boerman OC (2001). Factors affecting the accelerated blood clearance of polyethylene glycol-liposomes upon repeated injection. J. PharmacolExpTher..

[28] Ichihara M, Shimizu T, Imoto A (2010). Anti-PEG IgM Response against PEGylated Liposomes in Mice and Rats. Pharmaceutics.

[29] Ishida T, Ichihara M, Wang X (2006). Injection of PEGylated liposomes in rats elicits PEG-specific IgM, which is responsible for rapid elimination of a second dose of PEGylated liposomes. J. Control Release.

[30] Donini M, Pettinella F, Zanella G (2023). Effects of magnetic nanoparticles on the functional activity of human monocytes and dendritic cells. Int. J. Mol. Sci..

[31] Dwivedi PD, Tripathi A, Ansari KM, Shanker R, Das M (2011). Impact of nanoparticles on the immune system. J. Biomed. Nanotechnol..

[32] Yang EJ, Kim S, Kim JS, Choi IH (2012). Inflammasome formation and IL-1β release by human blood monocytes in response to silver nanoparticles. Biomaterials..

[33] Meunier E, Coste A, Olagnier D (2012). Double-walled carbon nanotubes trigger IL-1β release in human monocytes through Nlrp3 inflammasome activation. Nanomedicine.

[34] Dai T, Li N, Liu L, Liu Q, Zhang Y (2015). AMP-conjugated quantum dots: Low immunotoxicity both in vitro and in vivo. Nanoscale Res. Lett..

[35] Zhu M, Tian X, Song X (2012). Nanoparticle-induced exosomes target antigen-presenting cells to initiate Th1-type immune activation. Small.

[36] Park EJ, Oh SY, Lee SJ (2015). Chronic pulmonary accumulation of iron oxide nanoparticles induced Th1-type immune response stimulating the function of antigen-presenting cells. Environ. Res..

[37] Karlsson HL (2010). The comet assay in nanotoxicology research. Anal. Bioanal. Chem..

[38] Mai T, Hilt JZ (2017). Magnetic nanoparticles: Reactive oxygen species generation and potential therapeutic applications. J. Nanopart. Res..

[39] Yarjanli Z, Ghaedi K, Esmaeili A, Rahgozar S, Zarrabi A (2017). Iron oxide nanoparticles may damage to the neural tissue through iron accumulation, oxidative stress, and protein aggregation. BMC Neurosci..

[40] Fu PP, Xia Q, Hwang H-M (2014). Mechanisms of nanotoxicity: Generation of reactive oxygen species. J. Food Drug. Anal..

[41] Bardestani A, Ebrahimpour S, Esmaeili A, Esmaeili A (2021). Quercetin attenuates neurotoxicity induced by iron oxide nanoparticles. J. Nanobiotechnol..

